# Transforming Cardiology with AI: Ambient AI scribes in Epic

**DOI:** 10.1007/s12471-026-02089-9

**Published:** 2026-10-12

**Authors:** Fleur M.M. M M Meijer, Jonas S.S.G. S S G de Jong

**Affiliations:** Department of Cardiology, OLVG, Amsterdam, The Netherlands

## Introduction

Documentation has become a defining administrative burden in cardiology. In ambulatory practice, physicians spend a full day with one eye on the EHR and the other on the patient. Ambient artificial intelligence (AI) scribes promise to convert typing into a passive, voice-driven background process: a microphone captures the consultation, automatic speech recognition transcribes it, and a large language model (LLM) drafts a structured note that the clinician reviews and signs. Several vendors (Microsoft DAX Copilot/Nuance, Autoscriber) offer such solutions, and Epic’s own native ambient feature is now integrated directly into the Epic Hyperdrive client. OLVG was the first hospital in mainland Europe to adopt Epic’s ambient AI scribing at an early stage.

## Technical features and capabilities

**Fig. 1:**
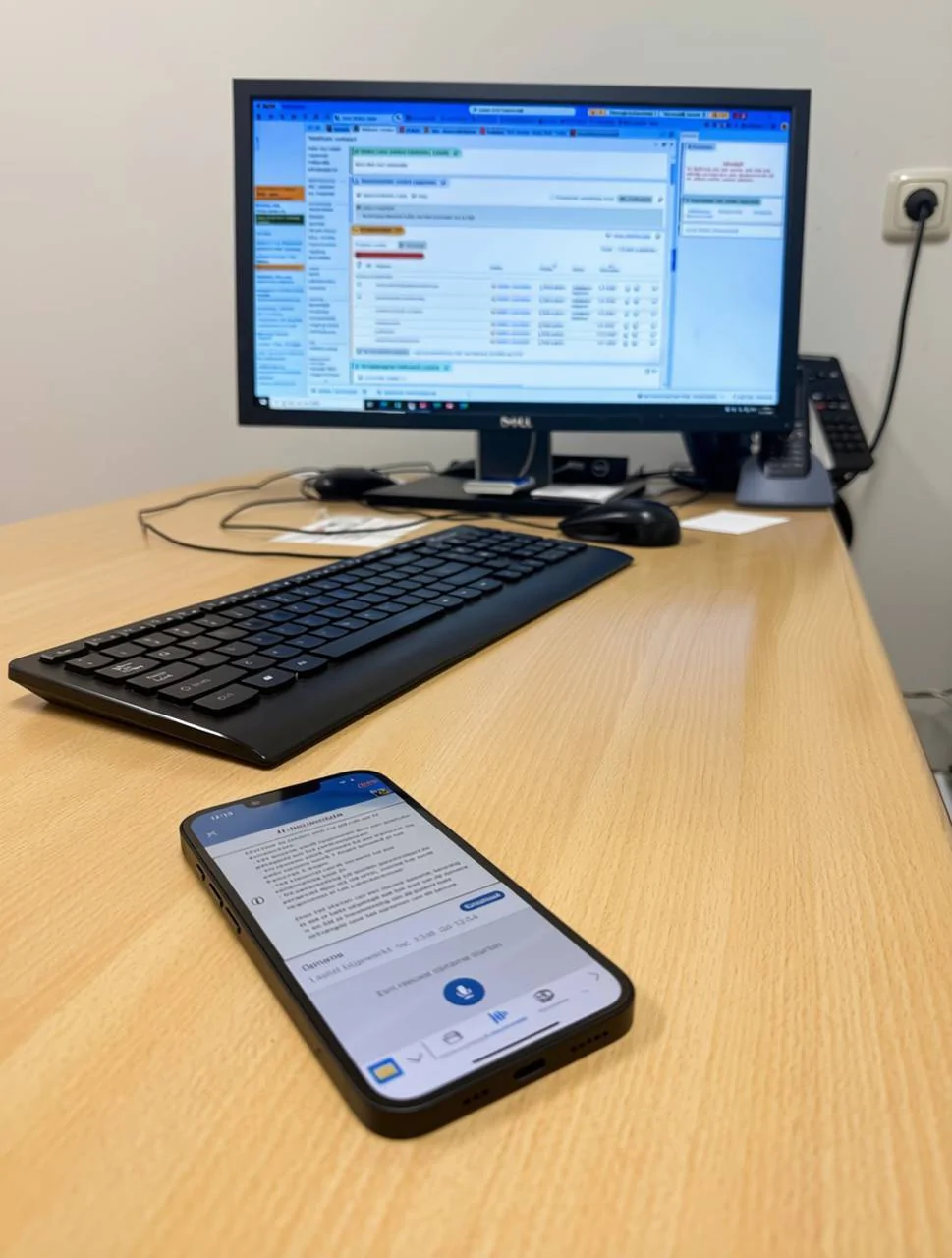
Haiku / EPIC installed on the smartphone transforms the phone into a microphone. The audio file is uploaded and the transcription appears in the note in EPIC on the desktop

In Epic, the workflow starts from the patient’s chart. The desktop shows the usual Epic screen. Epic Haiku is installed on a smartphone and the phone is only used as a microphone in this setup (Fig. 1). After obtaining brief consent, for example “*Are you ok for the computer to listen in so I don’t have to type?”,* a click on the phone starts an audio recording of the conversation. At the end of the session, the audio recording is uploaded automatically and after 15–60 s the progress note is pre-populated in Epic. It may also generate suggested orders via Epic’s “Art” interface. Audio recordings are deleted after 1 week once the note is produced.

Crucially, an Epic-integrated ambient scribe can do far more than transcribe the conversation. Much of what matters in a cardiology visit is never spoken aloud: the active medication list with doses, lab results, prior echocardiography, angiography and device interrogation reports, the problem list and prior letters all live in the chart.

With access to the chart, the model can ground its draft in the record: a spoken “diuretic” can be reconciled with the documented prescription of furosemide 20 mg once daily. A standalone recorder, working only from the conversation, cannot do this. This matters most in cardiology, where medication lists are long, decisions hinge on lab values and prior imaging, and patients are often co-managed with other specialties. Here Epic’s chart integration gives it a clear advantage over its ambient voice competitors.

## Potential clinical applications

The first thing we noticed during use was that ambient scribing genuinely shifted attention back to the patient: eye contact and active listening improved immediately, because the keyboard was no longer in the way. Epic Ambient AI fits the rhythm of structured cardiology follow-up visits, including atrial fibrillation management, heart failure titration, post-myocardial infarction review and valvular disease surveillance, where vocabulary is predictable and the format recurs. In the largest published implementation, more than 300,000 encounters were assisted in ten weeks, with physicians reporting more meaningful interactions and less after-hours work [1]; a multicentre US evaluation found that self-reported burnout dropped significantly within 30 days [2]. In our cardiology clinic, we saw about 30% less EPIC-related documentation time per outpatient visit in the first evaluation.

## Clinical considerations

The model makes non-human errors: we saw *pantoprazole* transcribed as *plantenzorg* (plant care)—a substitution no human scribe would make; and *nebivolol* as ‘patient rapporteert geen lol’ (patient reports no fun). Medication capture is inconsistent, sometimes pixel-perfect, sometimes plainly wrong. The clinician remains fully responsible for what is signed.

The drafts can also wander outside the cardiologist’s scope: we once caught a line reading “Advice: continue chemotherapy” after a patient had described their oncology history. The drafted history of present illness is often too long-winded, producing a dense block of text that is hard to scan. Putting the key elements in bold, such as the medication, decisions, and the follow-up, would make the note considerably easier to read. Some of these issues are easy to fix. Note quality scales with chart context: more is better. As specialists, we tend to sign off on junior doctors’ notes after only a quick scan; with AI-generated drafts, we found that habit unsafe—every line needs to be read before signing. It requires a new skill to become wary of the typical AI mistakes and hallucinations that are easily overread because such mistakes are unexpected.

## Future perspectives

Ambient AI scribing has already improved both clinician well-being and patient encounters from the first day of use. In practice it is useful, provided every note is read—not skimmed—before signing. In our experience this somewhat prolongs the time between encounters as you need to wait for the transcription and check it between encounters. The real leverage lies in deeper integration: feeding the model more structured Epic records is the next major gain. Integrating all this information into a well-structured, well-formatted note is a realistic next step.
